# New-Onset Autoimmune Diabetes Mellitus Presenting As Diabetic Ketoacidosis in Association With Pembrolizumab Therapy and Long Term Follow-Up: Case Report

**DOI:** 10.7759/cureus.24479

**Published:** 2022-04-25

**Authors:** Vrushali Pachpande, Sanjana Mullangi, Manidhar Reddy Lekkala, Arpan Patel

**Affiliations:** 1 Internal Medicine, Guthrie Medical Group, Sayre, USA; 2 Internal Medicine, University of Kansas Medical Center, Kansas City, USA; 3 Medical Oncology, University of Kansas Medical Center, Kansas City, USA; 4 Medical Oncology, University of Rochester Medical Center, Rochester, USA

**Keywords:** immunotherapy, immune checkpoint inhibitors, irae, immune-related adverse effects, pembrolizumab

## Abstract

Pembrolizumab, an immune checkpoint inhibitor (ICI) that acts against receptor programmed cell death-1 (PD-1), is currently being used in the treatment of a variety of cancers. As PD-1 is also present on other non-malignant tissues, this results in side effects involving a multitude of organ systems termed immune-related adverse effects (irAEs). Programmed cell death-1 is expressed on the beta cells of islets of the pancreas, and their destruction can result in hyperglycemia and the onset of new diabetes mellitus (DM). Thus, the anti-PD1 action of pembrolizumab can lead to autoimmune-related DM. We present a case of a 62-year-old male who developed new-onset DM after 12 cycles of pembrolizumab with a severe presentation in the form of diabetic ketoacidosis (DKA) and ICU stay. Our case underscores the importance of physician awareness, frequent lab monitoring and patient education about this rare but potentially fatal irAE of ICI. It also strengthens existing data in literature suggesting the association of irAEs with improved efficacy of ICI therapy.

## Introduction

Programmed cell death-1 (PD-1) is a transmembrane protein that acts as a receptor [1]. It is expressed on T cells and is activated by two ligands: PD-L1 and PD-L2. When PD-1 binds to PD-L1, an inhibitory signal is generated which regulates T-cell activation, tolerance, and cytotoxic activity. It can lead to suppression of the immune system by inducing apoptosis of T cells. In physiologic states, this prevents autoimmunity and essentially, tells the body not to attack itself. However, in a cancerous state, tumors create an immunosuppressive microenvironment by activating this inhibitory pathway, leading to a phenomenon known as T cell exhaustion. Pembrolizumab, from the class of immune checkpoint inhibitors (ICI), is a highly selective humanized monoclonal antibody against PD-1. It blocks the PD-1 pathway which can rescue the T cells from exhaustion and reinvigorate them, thus inducing an anti-neoplastic effect. However, PD-1 is not selectively expressed on T cells and is also present on other cells including hematopoietic cells, vascular endothelial cells, and pancreatic islet cells [2]. Thus, the action of pembrolizumab on PD-1 expressed on the non-malignant cells leads to a host of side effects termed immune-related adverse effects (irAEs).

Type 1 diabetes mellitus (DM) is caused by the destruction of insulin-producing β-cells of islets of Langerhans in the pancreas. It is further categorized as type 1A when the destruction is secondary to autoimmunity and type 1B when no evidence of autoimmunity can be found [3]. Several autoantibodies have been implicated in the pathogenesis of type 1A DM. Both PD-1 and PD-L1 blockade has been shown to precipitate DM in prediabetic nonobese diabetic (NOD) mice [4]. This mechanism may be underlying the pathogenesis of DM in patients receiving anti-PD-1 drugs. A few cases have been reported of a new onset of type 1 DM and at least 1 case of worsening glycemic control in a pre-existing type 2 DM patient receiving immune checkpoint inhibitors [5-8].

This case report aims to create awareness regarding this rare but potentially fatal complication of a promising branch of cancer immunotherapy, and perhaps shed light on outcomes thereafter.

## Case presentation

A 62-year-old Caucasian male presented with a past medical history of diet-controlled hypertension, deep vein thrombosis on anticoagulation, chronic obstructive pulmonary disease not on supplemental oxygen, benign prostate hyperplasia, dyspepsia, history of tobacco abuse with 35 pack-years smoking history, and notably no history of DM with him or his family. Home medications included Apixaban, Esomeprazole, Albuterol inhaler, and Finasteride. His last glycohemoglobin A1C checked about one year before presentation, was normal at five. During workup for shoulder pain, axillary lymphadenopathy was noted incidentally on imaging and a subsequent biopsy revealed adenocarcinoma of the lung with a high tumour proportion score (TPS) that was 60%. Staging scans showed multiple mediastinal and bilateral axillary necrotic lymph nodes as well as a soft tissue chest wall mass with adrenal gland metastasis. The patient was enrolled in a clinical trial and received single-agent chemotherapy with pemetrexed and immunotherapy with pembrolizumab with standard dosing every three weeks. He received 12 cycles of pembrolizumab without obvious idiopathic angioedema (iAE) during routine follow-up, and scans showed a reduction of disease burden by 80% by response evaluation criteria in solid tumors (RECIST) criteria and eventually progressing to no evidence of disease. Random non-fasting blood glucose on a routine metabolic panel up to six months before the current presentation ranged between 80 to 140 mg/dl. About two weeks after his twelfth cycle of pembrolizumab, the patient reported symptoms of vomiting, non-bloody diarrhea, excessive thirst, and polydipsia for four days. He had no improvement with anti-emetics and was referred to the emergency department.

During initial triage and workup, he was noted to be afebrile, tachycardic, and normotensive. Blood work revealed dramatic hyperglycemia and metabolic acidosis. His urinalysis showed no evidence of infection, however, it was suggestive of diabetic ketoacidosis (DKA) with significant glucosuria and ketonuria. The C-peptide levels were low, and autoantibodies i.e., islet cell and the 65-kDa glutamic acid decarboxylase enzyme (GAD65), were negative. Please refer to Table 1 for laboratory values.

**Table 1:** Laboratory parameters at initial workup pCO2: partial pressure of carbon dioxide, pH: Potential of hydrogen

Laboratory test	Result	Reference range
Sodium	121 mmol/L	136-145 mmol/L
Potassium	5.4 mmol/L	3.5-5.1 mmol/L
Chloride	84 mmol/L	98-107 mmol/L
Bicarbonate	12 mmol/L	22-29 mmol/L
Anion Gap	25 mmol/L	3-11 mmol/L
Creatinine	0.91 mg/dl	0.51-0.95 mg/dl
Glucose	600 mg/dl	70-99 mg/dl
Calcium	11.3 mg/dl	8.6-10 mg/dl
Serum beta-hydroxybutyrate	7.01 mmol/L	0.02 - 0.27 mmol/L
C-peptide	0.9 ng/mL	1.1-4.4 ng/mL
Venous blood gas		
pH	7.28	7.31-7.41
pCO2	26 mmHg	41-51 mmHg

The patient was diagnosed with new-onset DM and DKA. He was managed in the ICU with an intravenous insulin drip per DKA protocol, subsequently transitioned to subcutaneous insulin injections and transferred to the general medical floor. He was discharged with subcutaneous insulin and outpatient follow-up with endocrinology. On follow-up, the patient’s cancer is in remission, scans show no evidence of disease with an Eastern Cooperative Oncology Group (ECOG) performance status of 0, and he is on active surveillance. Pembrolizumab therapy was not restarted since the DKA at the time of this report. His DM is well controlled with insulin.

## Discussion

As our understanding of the role of the immune system in cancer treatment evolves, the indications for immunotherapy with systemic cancer treatment options widen. Immune checkpoint inhibitors were first approved by the FDA for melanoma in 2014 followed by non-small cell lung cancer (NSCLC) in 2015. Currently, they are also approved for use in urothelial carcinoma, squamous cell carcinoma of the head and neck, Hodgkin’s lymphoma etc. Pembrolizumab was the first ICI to receive FDA approval [9]. Due to the presence of the molecular targets of these drugs on normal tissues and the non-specificity of the action, several side effects can arise, named irAE as defined earlier. Among these, the development of type 1 DM is estimated in approximately 0.2% (pembrolizumab) to 0.9% (nivolumab) of cases [10]. The exact mechanism of development of autoimmune DM in patients treated with ICI is being elucidated. The time of onset after administration of the immunotherapy is variable. In a meta-analysis by Arturk et al., time to type 1 DM onset did not correlate to hemoglobin A1c (HbA1c), implying a short timeframe of significantly elevated blood glucose before onset. Type 1 DM is generally but not always attributed to increased genetic risk and the presence of antibodies [11]. In cases precipitated by immunotherapy, high-risk human leukocyte antigen genotypes were found in several patients and antibodies relevant to traditional type 1 DM predominantly anti-glutamic acid decarboxylase antibodies were found in slightly more than half of the cases [11-12]

Management recommendations are outlined in the American Society of Clinical Oncology (ASCO®) 2018 guidelines [13]. Patients receiving ICI therapy should be monitored for signs and symptoms of hyperglycemia indicating new-onset DM or worsening glycemic control of previously known DM. It is recommended to measure serum glucose before initiation of treatment to establish a baseline and subsequently with each cycle during the induction phase and then three to six weeks thereafter. Typically, a metabolic panel done every three weeks captures this. Management should be customized for each patient according to their medical comorbidities, and risk factors for the development of DM. In patients suspected to have developed new or worsening DM, workup should include testing for ketonuria or ketonemia and anion gap to diagnose DKA. Further information like the presence of autoantibodies, and C-peptide levels may assist in identifying the specific type of DM. Insulin therapy is the mainstay of the treatment. The decision to continue or withhold ICI depends on the grading based on the severity of the clinical presentations [13]. Unlike other irAEs, immunosuppression with glucocorticoids or infliximab is not beneficial in ICI-induced DM. On the contrary, glucocorticoids may worsen hyperglycemia in these patients [14]. Patients should be educated about the signs and symptoms of hyperglycemia and DKA, along with monitoring blood glucose. 

Our patient experienced a grade 4 irAE and a favourable outcome. This is in accordance with studies showing a correlation between the development of irAE with improved efficacy of ICI therapy and outcomes, suggesting irAE appearance to be a clinical biomarker for ICI response, especially if they are of severe grade or involve multiple systems [15-17]. This may indicate that the irAEs are unavoidable and inextricably linked with the clinical benefit of ICI therapy making their early identification and optimal treatment vitally important. 

## Conclusions

With the advent and widespread use of ICI for several types of cancers, a peculiar set of side effects termed irAEs has been described. We presented a case of a 62-year-old male patient with a particularly severe presentation of one of the irAEs in the form of DKA. Frequent monitoring of laboratory parameters, high degree of suspicion of development of irAEs and patient education are paramount to early diagnosis and treatment to prevent critical outcomes.
